# Commensal microbiota modulates phenotypic characteristics and gene expression in piglet Peyer’s patches

**DOI:** 10.3389/fphys.2023.1084332

**Published:** 2023-03-22

**Authors:** Jinwei Zhang, Yang Shen, Guitao Yang, Jing Sun, Chuang Tang, Hao Liang, Jideng Ma, Xiaoqian Wu, Haoran Cao, Meng Wu, Yuchun Ding, Mingzhou Li, Zuohua Liu, Liangpeng Ge

**Affiliations:** ^1^ Chongqing Academy of Animal Sciences, Chongqing, China; ^2^ Key Laboratory of Pig Industry Sciences, Ministry of Agriculture, Chongqing, China; ^3^ National Center of Technology Innovation for Pigs, Chongqing, China; ^4^ Yangling Food Engineering Innovation Center, Yangling, Shanxi, China; ^5^ Farm Animal Genetic Resource Exploration and Innovation Key Laboratory of Sichuan Province, Sichuan Agricultural University, Chengdu, Sichuan, China

**Keywords:** commensal microbiota, germ-free, specific pathogen-free, Peyer’s patches, phenotypic characteristics, mRNA, lncRNA

## Abstract

The gastrointestinal tract contains a complex microbial community. Peyer’s patches (PPs) play an important role in inducing mucosal immune responses in the gastrointestinal tract. However, little is known about the effect of commensal microbiota on the host’s PPs. Here, we analyzed the phenotypic-to-transcriptome changes in the intestine PPs of specific pathogen-free (SPF) and germ-free (GF) piglets (fed in an environment with and without commensal microbiota, respectively) to elucidate the role of commensal microbiota in host intestine mucosal immunity. Analyses of anatomical and histological characteristics showed that commensal microbiota deficiency led to PP hypoplasia, especially regarding B and T cells. A total of 12,444 mRNAs were expressed in 12 libraries; 2,156 and 425 differentially expressed (DE) mRNAs were detected in the jejunal PP (JPP) and ileal PP (IPP), respectively (SPF vs. GF). The shared DE mRNAs of the JPP and IPP were mainly involved in basic physiological and metabolic processes, while the specific DE mRNAs were enriched in regulating immune cells in the JPP and microbial responses and cellular immunity in the IPP. Commensal microbiota significantly modulated the expression of genes related to B-cell functions, including activation, proliferation, differentiation, apoptosis, receptor signaling, germinal center formation, and IgA isotype class switching, particularly in the JPP. TLR4 pathway-related genes were induced in response to microbial colonization and in LPS/SCFA-treated B cells. We also detected 69 and 21 DE lncRNAs in the JPP and IPP, respectively, and four one-to-one lncRNA-mRNA pairs were identified. These findings might represent key regulatory axes for host intestine mucosal immunity development during microbial colonization. Overall, the findings of this study revealed that commensal microbiota modulated phenotypic characteristics and gene expression in the piglet intestine PPs and underscored the importance of early microbial colonization for host mucosal immunity development.

## 1 Introduction

Mammalian mucosal tissues are immediately exposed to the environment after birth and colonized by commensal microbiota, such as bacteria, fungi, and viruses (Spasova and Surh, 2014). Over 100 trillion microbes inhabit the gastrointestinal tract and form a symbiotic relationship with the host, which is critical for the homeostasis and health of the host’s gut (He et al., 2021). The gut is critical for the digestion and absorption of nutrients and is an important immune site that tolerates exogenous antigens in the intestinal lumen (e.g., commensal microbiota and food) while protecting the host from foreign pathogens (Olivares-Villagómez and Van Kaer, 2018). Peyer’s patches (PPs) are critical for capturing, processing, and presenting antigens and play an important role in inducing mucosal immune responses in the gastrointestinal tract. Foreign antigens, like commensal microbiota, enter PPs *via* microfold (M) cells and are processed by antigen-presenting cells, such as dendritic cells and macrophages, and presented as MHC class II antigen-derived peptides. Specific T cells are activated by foreign antigens and stimulate B cells, initiating a mucosal immune response and leading to the production of specific antibody immunoglobulin A (IgA) (Tamburini et al., 2016; Furukawa et al., 2020). IgA is transported to the intestinal lumen in the form of secretory IgA (SIgA) through the transcytosis of intestinal epithelial cells *via* the polymeric immunoglobulin receptor (pIgR). SIgA then selectively binds to antigens on the pathogen’s surface, forms a specific immune complex, and neutralizes or eliminates the pathogen (Brandtzaeg, 2002).

Pigs are anatomically, physiologically, and metabolically similar to humans and have been used as indispensable animal models in life science and medical research (Meurens et al., 2012; Lunney et al., 2021). Notably, the size, shape, and developmental location of PPs differ between species (Liebler-Tenorio and Pabst, 2006; Mörbe et al., 2021). There are two types of PPs in pigs, the jejunal PP (JPP) (scattered distribution) and the ileal PP (IPP) (continuous distribution) (Levast et al., 2010). The development of porcine IPPs is initiated prenatally on the 76th day of embryogenesis. Porcine IPPs gradually mature with age and an increase in antigen stimuli, then begin to degrade at 5 years of age until they become absent (Pabst et al., 1988; Furukawa et al., 2020). Porcine JPPs develop from the 50th day of embryogenesis and remain throughout the animal’s life (Liebler-Tenorio and Pabst, 2006). IPPs and JPPs grow rapidly during the 10 days before birth and the first weeks after birth (Liebler-Tenorio and Pabst, 2006). There is a significant difference in the proportion of immune cells between JPPs and IPPs. For example, T cells are mainly located in the interfollicular region (IFR) in the JPP, while the proportion of germinal center (GC) B cells in the follicular region (FOR) is higher in the IPP (Maroilley et al., 2018). Furthermore, the results of a previous study revealed that IPP resection in newborn piglets did not result in obvious changes in the proportion of T and B cell subsets, suggesting that IPP is non-essential for systemic B cell development and maintenance in piglets (Sinkora et al., 2011).

**FIGURE 1:**
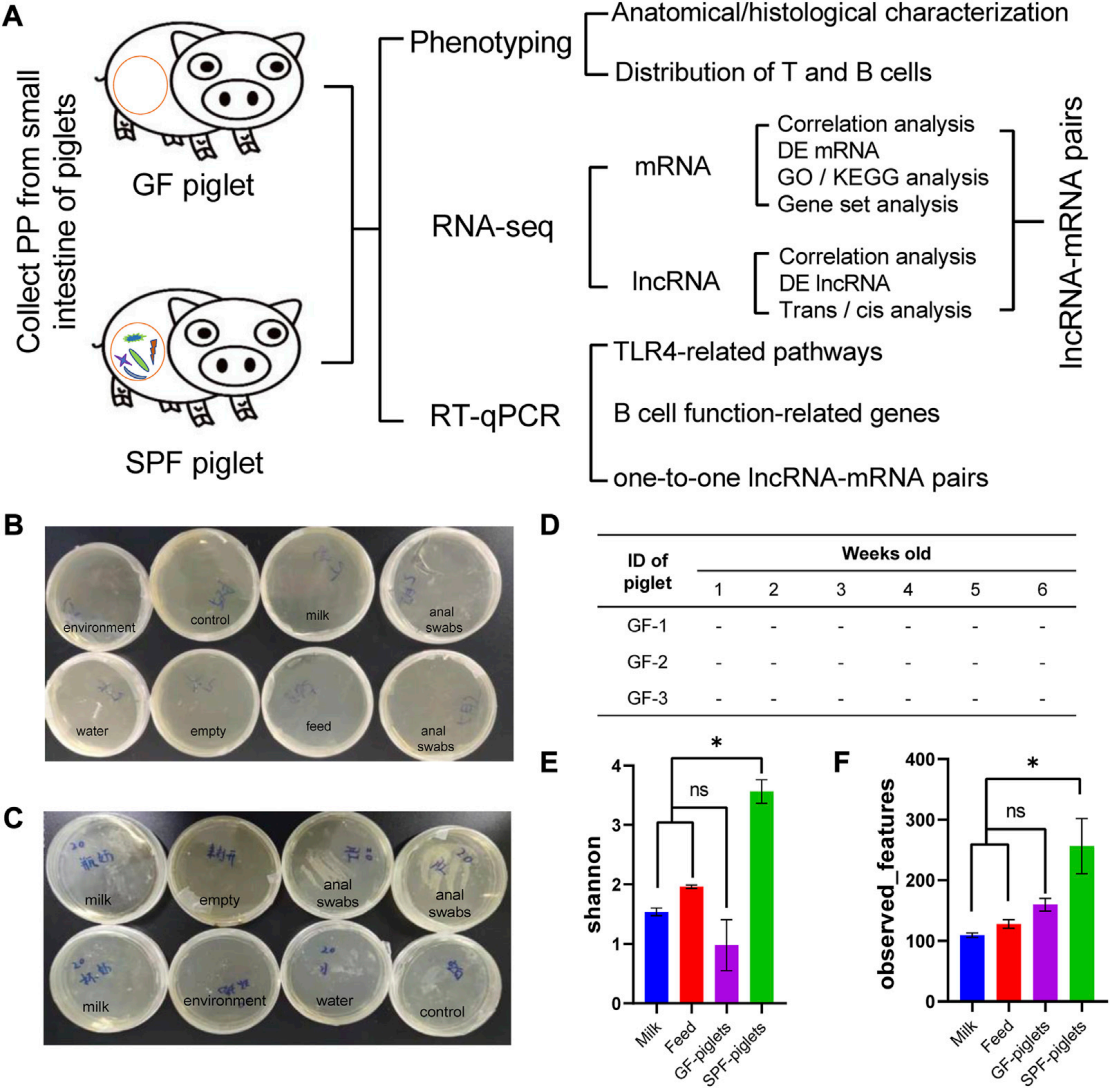
Schematic illustration of this study and the microbiota detection protocol. **(A)** Flow chart of PP phenotype and RNA-seq analyses. Medium samples were used to detect microbiota in the feeding isolator, including in the environment, feed, milk powder, and piglet anal swabs [Normal **(B)** and abnormal **(C)** detection]. **(D)** GF piglets remained sterile during the experiment (tested once a week). **(E,F)** Microbiota detection in feed, milk powder, and piglet anal swabs by 16s rDNA amplicon sequencing. Shannon and observed features indices reflect the α diversity of the microbiota. *n* = 3.

Many studies have revealed the regulatory effect of commensal microbiota on host immune tissues, such as the spleen, or on the immune cells of non-immune tissues, such as the skin (Meisel et al., 2018; Sun et al., 2018). However, few studies have been conducted on the role of commensal bacteria on host intestinal mucosal immunity in large animal models. Germ-free (GF) and specific pathogen-free (SPF) piglets are ideal large animal models for researching host–microbe interactions (Zhou et al., 2021a). In this study, we comprehensively analyzed the phenotypic-to-transcriptomic changes in the small intestinal PPs of SPF and GF piglets (Figure 1A) and revealed the importance of early microbial colonization for the development and maturation of host mucosal immunity, providing a new idea for studying host–microbe interactions and intestinal health.

## 2 Materials and methods

### 2.1 Ethics approval

The ethics committee of the Chongqing Academy of Animal Science reviewed the relevant ethical issues and approved this study (permit number XKY-No. 20210606). All animal experiments were conducted at the Experimental Swine Engineering Center of the Chongqing Academy of Animal Sciences (CMA No. 162221340234).

### 2.2 Animals

Three GF piglets were obtained by sterile hysterectomy from a multiparous Bama sow (a common experimental breed in China). Briefly, on 112 days of gestation (full-term, 114 days), the pregnant Bama sow was anesthetized with 4% isoflurane. The uterus was excised and transferred to a sterile isolator through an aqueduct containing 0.05% peracetic acid. The newborn piglets were then removed from the uterus and transferred to sterile rearing isolator (Class Biologically Clean Ltd., Madison, WI, United States) *via* a sterile transfer vehicle. A total of three SPF piglets were delivered by hysterectomy and raised in rearing isolators in an SPF air environment. Both SPF and GF piglets were fed Co60 γ-irradiated sterilized milk powder diluted with sterile water 1:4 (0–21 days) and pellet feed (21–42 days), as previously described (Zhou et al., 2021b). During feeding, milk powder, feed and water were introduced into the isolator through a transfer cartridge and sterilized with 1% peracetic acid to prevent microbial contamination. According to the Chinese National Standard (GB/T 14926-41-2001), the GF environments were checked *via* anaerobic (thioglycolic acid medium) and aerobic (brain–heart perfusion broth) culture at least once a week (Figures 1B–D). A rectal swab was evaluated using 16s rDNA amplicon sequencing at the end of the experiment to further confirm the sterile status of the animals (Figures 1E, F).

### 2.3 Sample collection

At 42 days of age, SPF and GF piglets were anesthetized with isoflurane and exsanguinated. The abdominal cavity was then opened in a sterile environment. The small intestinal segments were separated and ligated to distinguish the PP tissues (JPP and IPP), the contents were rinsed with PBS, and an incision was made along the opposite sidewall of the mesentery. The fresh PP tissues were imaged. A portion of the fresh tissue was fixed with 4% (w/v) paraformaldehyde (Beyotime, China) for histomorphology analysis, and the remaining samples were snap-frozen in liquid nitrogen and stored at −80°C until further analysis.

### 2.4 Hematoxylin–eosin (HE) and immunohistochemistry (IHC) staining

The fixed tissue was dehydrated and embedded in paraffin for HE staining. Tissue blocks were sliced, and the tissue sections (5 mm) were deparaffinized and stained with hematoxylin and eosin (Beyotime, China). B and T cell distributions were evaluated by IHC staining using a pig-specific marker. The sections were treated with REAL Target Retrieval Solution (DAKO, Germany) for 40 min at 98°C or with 0.05% (w/v) of proteinase (Sigma, United States) for 3 min at 37°C for antigen retrieval, then incubated with 0.5% (w/v) blocking reagent (PerkinElmer, United States) for 30 min at room temperature (RT) to block the non-specific binding of antibodies. The sections were treated with primary antibodies [rabbit anti-CD20 (1:100, Biocare Medical, United Kingdom), rabbit anti-CD3 (1:100, SP7, Abcam, United Kingdom)] overnight at 4°C followed by secondary antibodies for 1 h at RT. The stained sections were observed and imaged using an Olympus IX53 microscope (Olympus, Japan). The integrated optical density (IOD) value was determined using Image-Pro Plus (IPP) software.

### 2.5 16s rDNA amplicon sequencing and data analysis

Total genomic DNA from feed, milk, and colon content samples (SPF and GF piglets) were extracted using a DNA Isolation kit (QIAGEN, China), and quantified using a Qubit 2.0 (Invitrogen, United States). The V3-V4 hypervariable regions of the 16S rDNA gene were amplified by universal primers with barcodes. PCR products were purified with a GeneJET Gel Extraction Kit (Thermo Scientific, United States). Sequencing libraries were generated using a TruSeq DNA PCR-Free Library Preparation Kit (Illumina, United States) following the manufacturer’s recommendations and sequenced using the 250 bp paired-end strategy on an Illumina NovaSeq platform (Novogene, China). Sequencing data were analyzed using the QIIME2 software package (Quantitative Insights Into Microbial Ecology), and in-house R scripts were used to analyze alpha- (within samples) and beta- (among samples) diversity (Bolyen et al., 2019). The 16s rDNA-seq data have been deposited in the Genome Sequence Archive (GSA) with accession number CRA008620.

### 2.6 Total RNA extraction, library preparation, and sequencing

Total RNA was extracted from the PP tissues using a HiPure Total RNA Mini Kit (Magen, China) per the manufacturer’s instructions. Total RNA with a ratio of absorbance at 260/280 nm ranging from 1.8 to 2.0 and a RIN value >8 was selected for further study. A total of twelve RNA-seq libraries (SPF vs. GF, JPP and JPP, three replicates) were constructed and sequenced on the DNBSEQ-T7 seq platform (Novogene, China), and 150-bp paired-end reads were obtained. High-quality data (clean data) were generated from the raw data through rigorous quality control (removing poly-N and low-quality reads, including those with ≥10% N, >10 nt aligned to the adapter with ≤10% mismatches allowed, and with >50% of bases with phred quality <5). The RNA-seq data have been deposited in the GSA with the accession number CRA008456.

### 2.7 Expression analysis of mRNA and lncRNA

Clean data were aligned to the pig reference genome (Sscrofa11.1) by HISAT2 (v2.2.1) and quantified by featureCounts from the Rsubread package (v2.8.1). mRNA and lncRNA expression levels were quantified using kallisto software (v0.44.0), and the transcripts per kilobase of exon model per million mapped reads (TPM) value of each sample was calculated (Pertea et al., 2015). mRNAs and lncRNAs with TPM values >1 and >0.1, respectively, in at least three repeats within one group, were considered to be expressed. Then, edge R (Bioconductor version: Release 3.10) was used to identify differentially expressed (DE) mRNAs and lncRNAs. mRNAs with adjusted-*P* values <0.05 and |log2(FC)| > 1 were regarded as DE mRNAs (Smyth, 2010). Due to the large variation and low expression, lncRNAs with adjusted *p* values <0.01 and |log2(FC)| > 1 were regarded as DE lncRNAs.

### 2.8 DE mRNA functional enrichment and gene set analyses

Gene Ontology (GO) and Kyoto Encyclopedia of Genes and Genomes (KEGG) pathway functional enrichment analyses were performed on the DE mRNAs using Metascape (http://metascape.org/) (Zhou et al., 2019). Gene set analyses were executed to systematically dissect the changes in B cell function-related genes induced by commensal microbiota. The B cell function-related gene sets were referenced from the GO website and divided by 10 biological processes: B cell activation, B cell proliferation, B cell differentiation, B cell apoptosis, germinal center formation, plasma cells differentiation, B cell chemotaxis, B cell homeostasis, BCR signaling pathways, and isotype switching to IgA isotypes. These genes were often derived from human- or mouse-based studies, and this study only evaluated the genes that were one-to-one orthologs with the pig genome. The gene expression changes were visualized *via* heatmaps (log TPM).

### 2.9 LncRNA *cis*/*tans* analysis

Conjoint *cis*/*tans* analyses were performed to indirectly predict lncRNA functions (Jin et al., 2019). Briefly, the protein-coding genes within 100 kb of the DE lncRNAs were collected, and those among the DE mRNAs were filtered (*cis* analysis). Then, Hmisc (version 3.4.4.) was applied to calculate the Pearson correlations between lncRNAs and mRNAs; mRNAs with high correlations (|*r*| > 0.90 and *p* < 0.05) were collected (*trans* analysis). Finally, the results of *cis* and *trans* analysis were integrated to identify one-to-one lncRNA–mRNA pairs (Zhang et al., 2020).

### 2.10 Cell culture

Hmy2. cir, a human B lymphoblastoid cell line derived from late-stage B cell cells, was routinely cultured in Iscove’s Modified Dubecco’s Medium (IMDM) (YaJi Biological, China) supplemented with 10% fetal bovine serum (Sigma, United States), 100 U/mL penicillin, and 0.1 mg/mL streptomycin (Solarbio, China) in a humidified 5% CO_2_ atmosphere at 37°C. The cells were stimulated with lipopolysaccharides (LPS) (10 μg/mL) (Beyotime, China) or short-chain fatty acids (SCFA)–acetic acid (1 mM), propionic acid (1 mM) and butyric acid (0.1 mM) (Sangon Biotech, China). After reaching the determined time point, the cells or supernatant were collected for subsequent analyses.

### 2.11 Cell viability and LDH release assay

Cell viability and LDH release were measured by a CCK8 assay and the LDH Cytotoxicity Assay Kit (Beyotime, China), respectively, per the manufacturer’s instructions. A total of 1 × 10^5^ cells/well were incubated in 96-well plates for 72 h, and samples were taken at six time points (0 h, 12 h, 24 h, 36 h, 48 h and 72 h) for the cell viability and LDH release analyses. For the CCK8 assay, 10 μL CCK8 reagent was added to every well 4 h before detection, then optical density (OD) 450 nm values were measured by a microplate reader (Thermo Fisher Scientific, Spain). For the LDH release assay, the culture medium in each well was premixed with the relevant reagent and incubated according to the manufacturer’s instructions. OD490 nm values were then measured. At least six independent experiments were performed per group. All values are presented as the mean ± standard deviation (SD).

### 2.12 Enzyme-linked immunosorbent assay (ELISA)

Cell supernatants were used for cytokine detection by ELISA. Briefly, 2 mL of a 2 × 10^5^ cell suspension/well was plated into a 24-well flat-bottomed plate, and the cell supernatants were collected after stimulation with LPS/SCFA for 48 h. The cytokine (TNFα and IL-6) levels were measured by ELISA (j&l Biological, China) per the manufacturer’s instructions.

### 2.13 Real-time quantitative PCR (RT–qPCR)

Total RNA was extracted from the PP tissues and cell samples using a HiPure Total RNA Mini Kit (Magen, China). mRNA and lncRNA were reverse-transcripted from total RNA using the PrimeScriptTM RT Reagent Kit with gDNA Eraser (Takara, China). The RT–qPCR reaction system (in 10 µL) included 1 µL cDNA, 0.8 µL primers, 0.2 µL ROX Reference Dye, 5 μL TB Green Premix Ex Taq (TaKaRa, China), and 3 µL RNA-free water. The following PCR reaction was performed in a Quant Studio 6 Flex instrument (Thermo Fisher Scientific, United States): 95°C for 30 s, followed by 40 cycles of 95°C for 5 s and 60°C for 34 s, then a melting curve analysis (65°C–95°C). The amplification efficiencies neared 100%, and the relative expression levels of mRNA and lncRNA were calculated using the 2^-△△CT^ method and expressed as the fold change relative to the GF or cell control group (Livak and Schmittgen, 2001). *GADPH* was used to normalize the gene expression levels (Barber et al., 2005; Gaur et al., 2014; Li et al., 2019). The specific primer sequences in this study are listed in Supplementary Table S1-1.

### 2.14 Statistical analyses

All experiments were performed as at least three independent experiments with three technical replicates. The data conformed to the normal distribution tested by GraphPad prism software (Shapiro-Wilk test), and presented as the mean ± SD. Significance tests were performed using SPSS 22.0 software (SPSS, Chicago, United States). An unpaired Student’s t-test and a one-way ANOVA with Tukey’s *post hoc* test were used to analyze the differences between two groups or three or more groups, respectively. *p* < 0.05 was considered to represent significance (^*^ *p* < 0.05, ^**^ *p* < 0.01 and ^***^ *p* < 0.001).

## 3 Results

### 3.1 Commensal microbiota affects the anatomical and histological characteristics in piglet PPs

PPs are the hub of the intestinal mucosal immune response and have the typical anatomical structure of lymphoid tissue. We established SPF and GF piglet models to study the differences in small-intestine PPs between these two groups (Figure 1A). GF piglets were successfully prepared and identified by plate culture (Figures 1B–D) and 16s rDNA-seq methods (Figures 1E, F).

**FIGURE 2:**
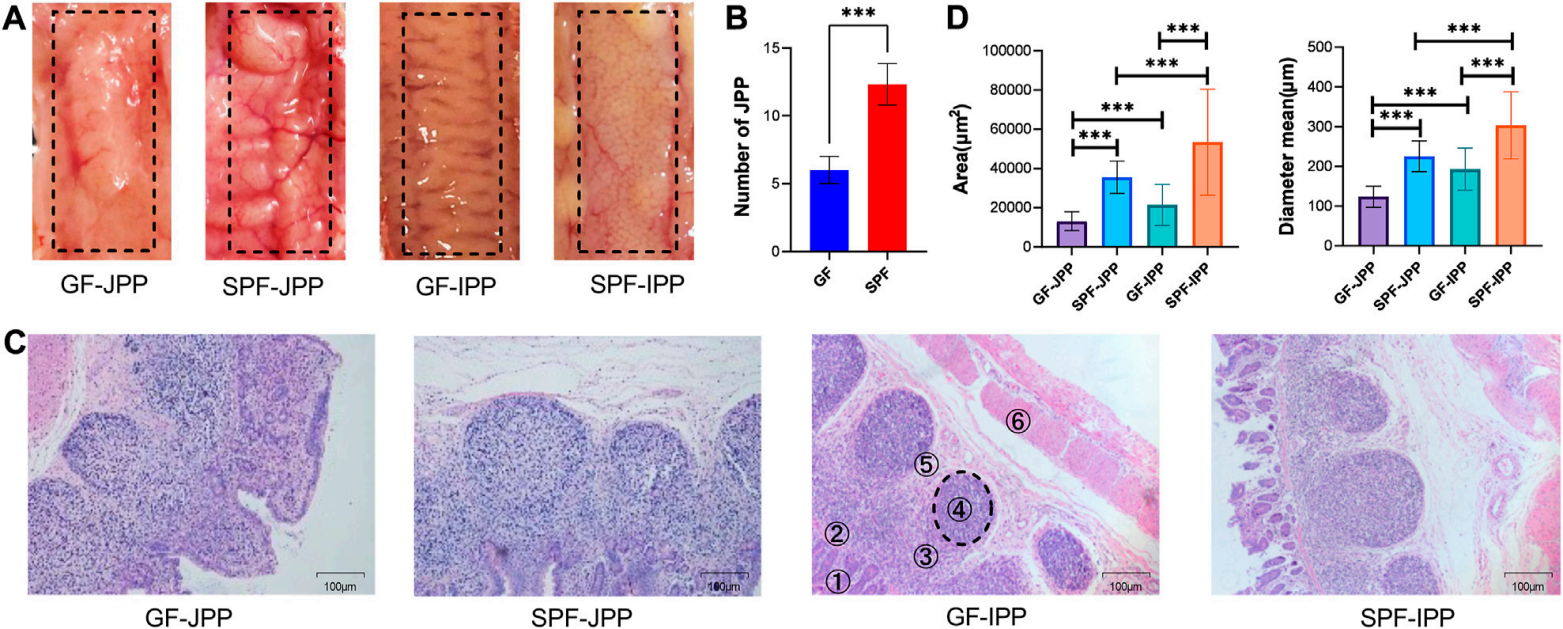
Anatomical and histological characteristics of PPs. **(A)** The anatomical characteristics of PPs observed by the naked eye. **(B)** Quantity statistics of JPPs. **(C)** The histological characteristics of PPs (1: villus lamina propria; 2: epithelial layer; 3: subepithelial dome area; 4: follicular area; 5: interfollicular area and 6: muscle layer); scale bar: 100 μm. **(D)** Quantitative analysis of the area and diameter of GC in PPs. Means ± SDs are presented for each group. *n* = 3. ^***^ *p* < 0.001.

The appearance of JPPs and IPPs were not obviously different between SPF and GF piglets (Figure 2A), and the number of JPPs was lower in GF piglets (*p* < 0.001) (Figure 2B). HE staining of PP tissues confirmed that the anatomical structure of the PPs was retained in both SPF and GF piglets (Figure 2C). The diameter and area of the GC of JPPs and IPPs in SPF piglets were significantly larger than those in GF piglets (*p* < 0.001); in both piglet groups, the diameter and area of the GC in IPPs were significantly larger than those in JPPs (*p* < 0.001) (Figure 2D). These results suggest that commensal microbiota promote the development of small-intestine PPs and that the GC in IPPs is better developed than that in JPPs.

### 3.2 Commensal microbiota influences the distribution of B and T cells in piglet PPs

**FIGURE 3:**
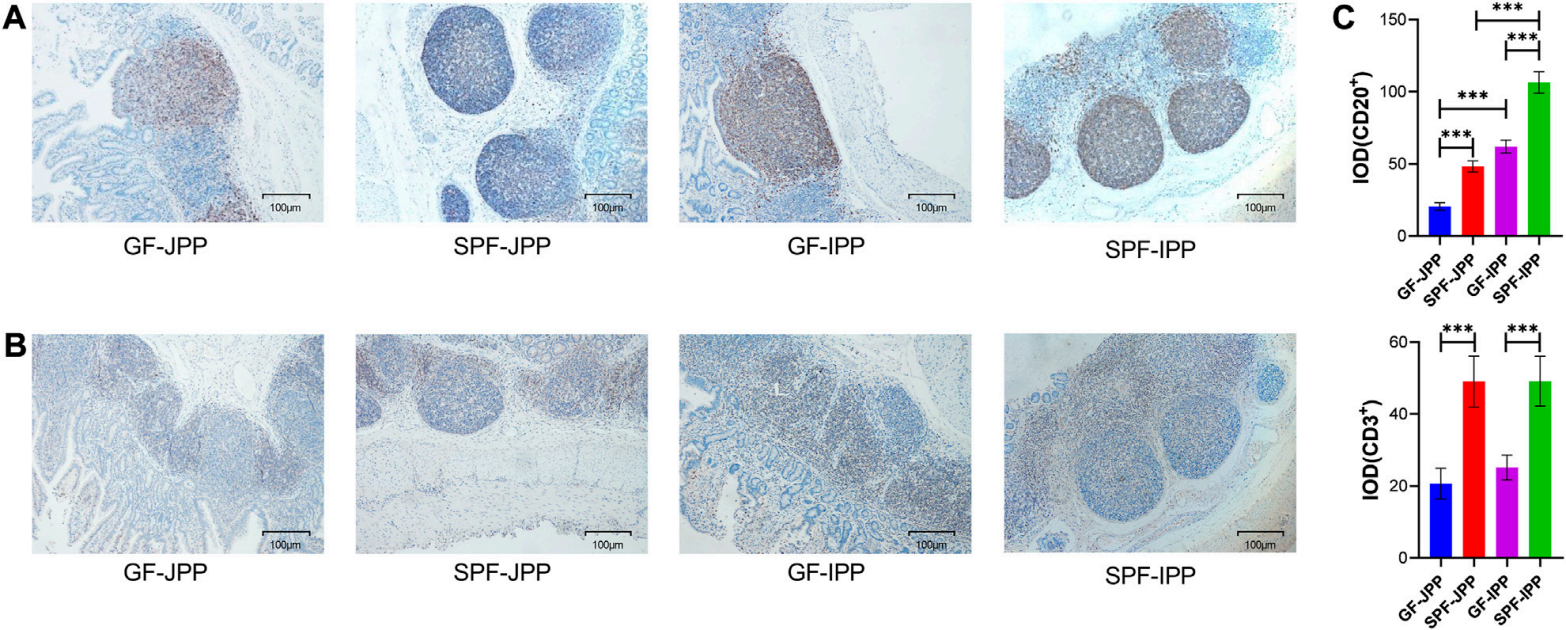
The distribution of B and T cells in intestinal PPs. The distribution of B and T cells detected by IHC staining for pig-specific CD20 **(A)** and CD3 **(B)** antibodies, respectively; brown indicates B/T cell specific staining; scale bar: 100 μm. **(C)** Quantitative analysis of the proportion of CD20^+^ and CD3^+^ cells. Means ± SDs are presented for each group. *n* = 3. ^***^ *p* < 0.001.

PPs contain various immune cells, especially B and T cells, which are crucial for the initiation and effect of mucosal immune responses. IHC staining of porcine-specific B and T cell markers revealed that B and T cells were mainly distributed in the GC in the FOR and the IFR, respectively, in IPPs and JPPs (Figures 3A, B). The proportion of B cells in the GC of SPF piglets was significantly higher than that in GF piglets (*p* < 0.001), and the proportion of T cells in the IFR demonstrated similar results (*p* < 0.001) (Figure 3C), indicating that the development of B and T cells in piglet PPs depends on continuous stimulation from the commensal microbiota. Interestingly, we found that regardless of whether the commercial microbiota is present, the proportion of B cells in IPPs was significantly higher than that in JPPs (*p* < 0.001), indirectly confirming that IPPs are important for the development and maturation of B cells in the small intestine.

### 3.3 Commensal microbiota regulates the mRNA expression of piglet PPs

**FIGURE 4:**
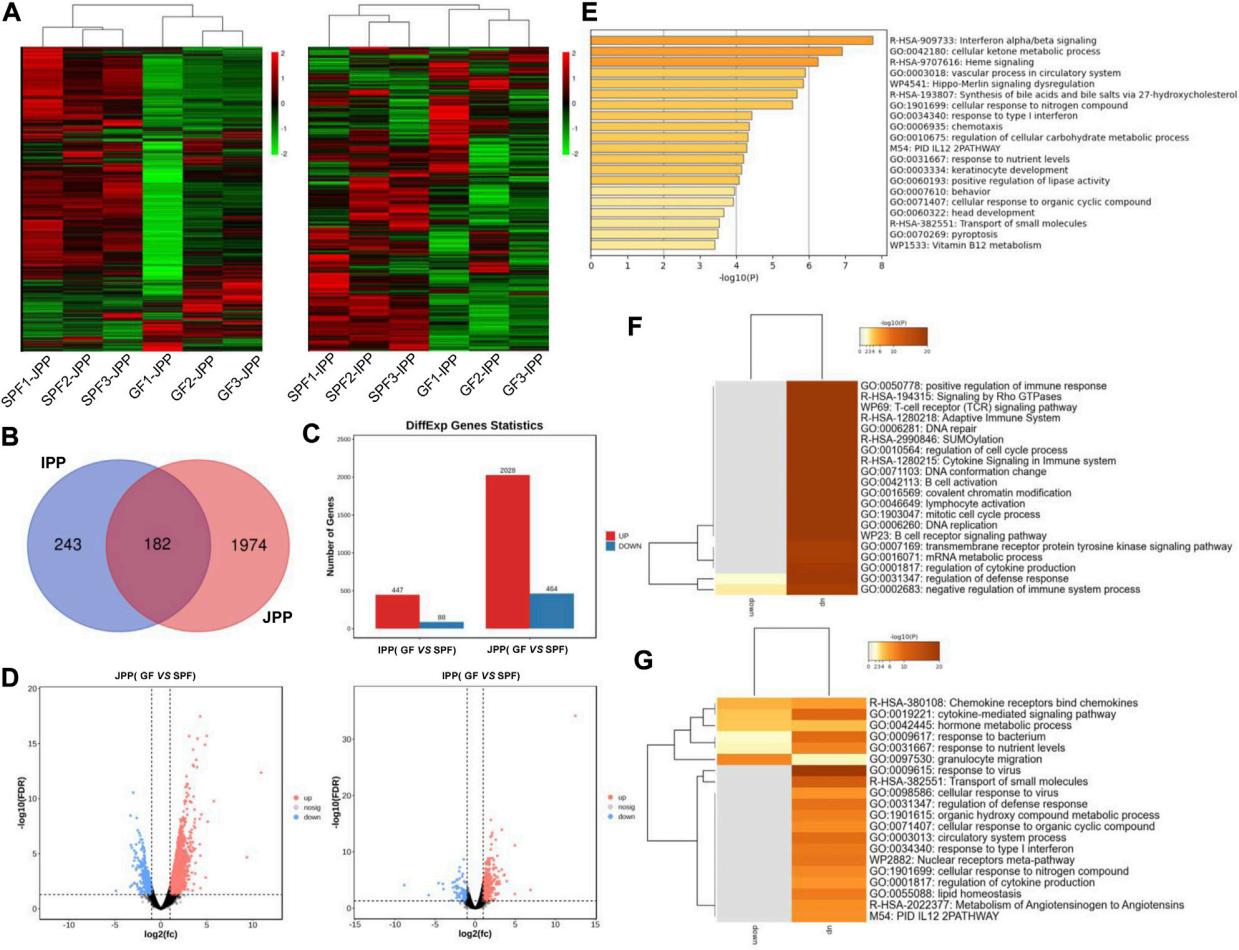
mRNA expression profiles of intestinal PPs in SPF and GF piglets. **(A)** Hierarchical clustering based on global expression profiles of mRNAs in IPPs or JPPs. **(B)** The number of shared and specific DE mRNAs in IPPs and JPPs. **(C)** The number of upregulated and downregulated DE mRNAs in JPPs and IPPs. **(D)** The DE mRNAs in JPPs and IPPs were analyzed and visualized by volcano plotting. **(E)** Functional enrichment analysis of shared DE mRNAs in JPPs and IPPs. Functional enrichment analysis of specific DE mRNAs in JPPs **(F)** and IPPs **(G)**. *n* = 3.

To explore the molecular mechanisms underlying the phenotypic differences of small-intestine PPs in the absence and presence of the commensal microbiota, RNA-seq was used to analyze the transcriptome changes in PPs in SPF and GF piglets. We obtained ∼216.25 G clean data from twelve libraries (≈18.28 G per library) (Supplementary Table S1-2), and a total of 12,444 mRNAs were expressed (TPM >1 at least three replicates of one group). Hierarchical clustering based on the overall expression in JPPs and IPPs showed that the three replicates per group clustered together (Figure 4A).

We analyzed DE mRNAs in twelve samples (SPF vs. GF) with the cutoff (|log2(FC)| > 1; FDR <0.05). In total, 2,492 (2,028 upregulated and 464 downregulated) and 535 (447 upregulated and 88 downregulated) DE mRNAs were obtained in JPPs and IPPs, respectively (Figures 4C, D; Supplementary Table S2). These results suggest that the commensal microbiota affected the gene expression profiles of PPs. The 182 DE mRNAs shared by JPPs and IPPs were mainly involved in the biological processes of cell activity, energy metabolism, and immune signal transduction, such as cell response to organic ring compounds, cell response to nitrogen compounds, ketone metabolism process, and regulation of cellular carbohydrate metabolism processes (Figures 4B, E). The 1,974 unique DE mRNAs in JPPs were enriched in the regulation of immune cells, for example, B cell receptor signaling, B cell activation, T cell receptor signaling pathway, and immune system cytokine signaling (Figure 4F). The 243 unique DE mRNAs in IPPs were enriched in microbial responses and cellular immunity, for example, responses to bacteria, responses to viruses, and responses to type I interferon (Figure 4G; Supplementary Table S3). These results suggest that the commensal microbiota influences basic cellular physiological and metabolic processes and immune functions. Interestingly, the intestinal immune network for IgA production was significantly enriched in PPs (Supplementary Figure S1). The above results indicate that the commensal microbiota induces gene expression changes in piglet PPs and that some of these genes are involved in host immune functions.

### 3.4 Commensal microbiota modulates the expression profiles of B cell function-related genes

**FIGURE 5:**
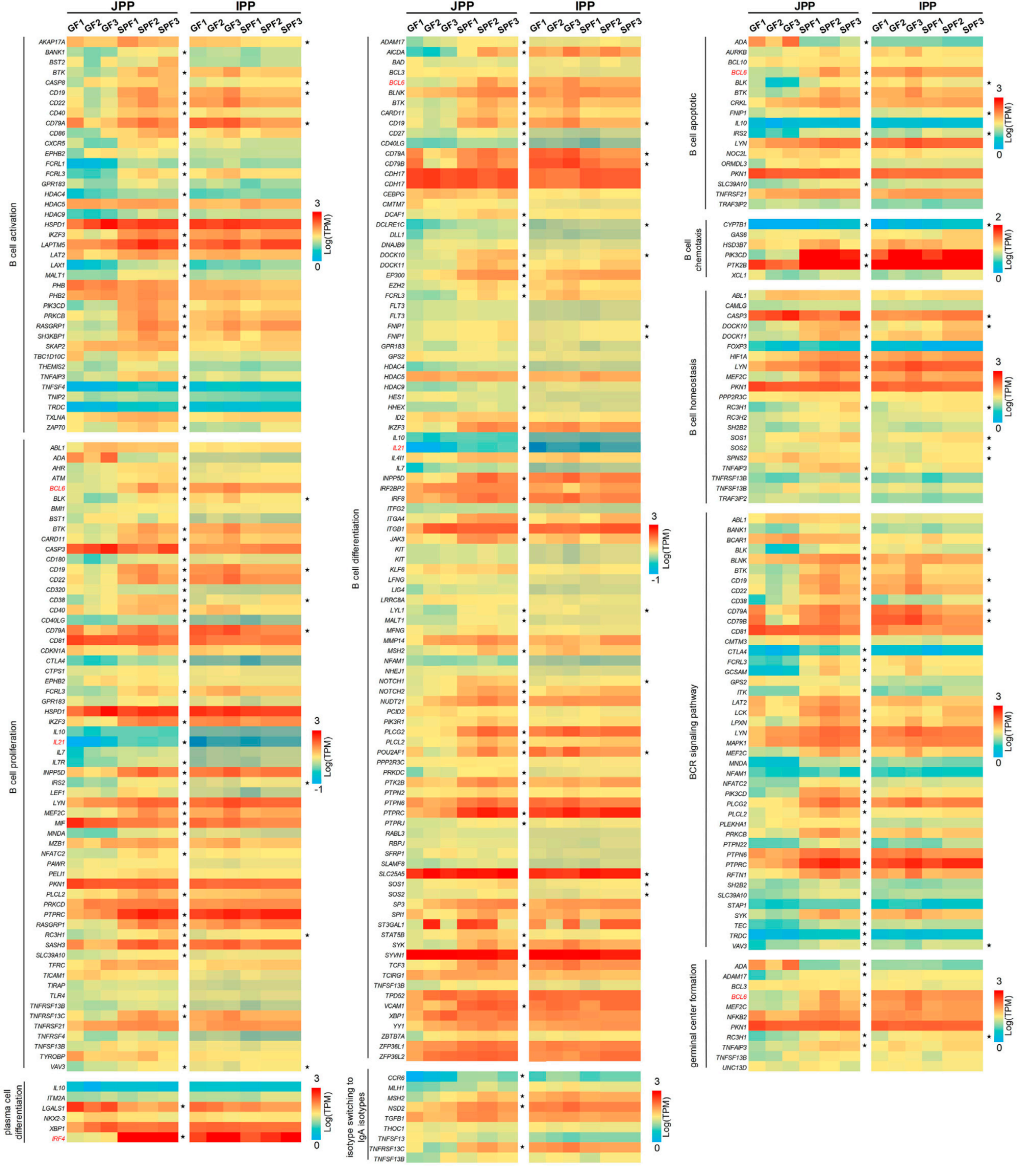
The expression profiles of B cell function-related genes. B cell function-related genes were involved in 10 biological processes. The expression levels of these genes (only one-to-one pig ortholog genes were used) are presented as a heatmap [Log(TPM)]. The genes marked in red (*IFR4*, *BCL6* and *IL21*) are mentioned in the “Discussion” section.

GC B cells in PPs are the main source of IgA production after antigen stimulation. The function of B cells is complex and related to many biological processes (Brandtzaeg, 2002). To study whether B cell functions are affected by the commensal microbiota, we compiled B cell function-related gene sets dividing genes by 10 biological processes: B cell activation, B cell proliferation, B cell differentiation, B cell apoptosis, germinal center formation, plasma cell differentiation, B cell chemotaxis, B cell homeostasis, BCR signaling pathways, and isotype switching to IgA isotypes. As shown in Figure 5, the commensal microbiota induced the expression of most B cell function–related genes in JPPs; the expression of 160 genes (50.80% of the total genes) was significantly upregulated. In contrast, the trend of expression profiles was weaker in IPPs (41 genes, accounting for 13.20% of the total) (Supplementary Table S4). These results indicate that the commensal microbiota affects intestinal mucosal immunity by modulating the expression of B cell function–related genes to different degrees.

### 3.5 Commensal microbiota affects the TLR4 signaling pathway

**FIGURE 6:**
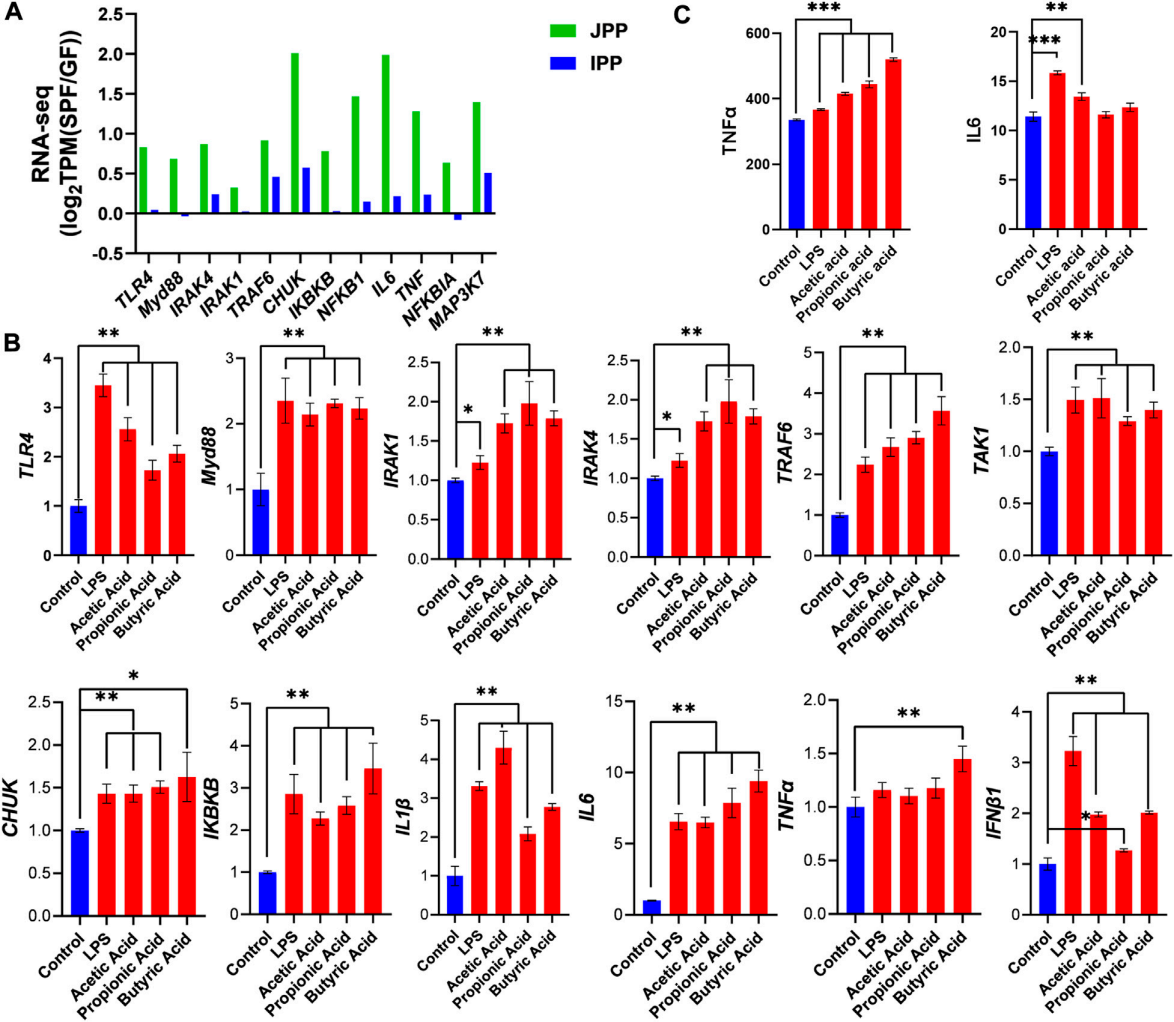
Commensal microbiota induces the TLR4 signaling pathway. **(A)** The expression of TLR4 signaling pathway-related genes in JPPs and IPPs [log_2_TPM(SPF/GF)]. The expression of TLR4 signaling pathway-related genes **(B)** and the secretion of IL6 and TNFα **(C)** were detected after 48 h stimulation with LPS or SCFAs (acetic acid, propionic acid and butyric acid) in Hmy2. cir cells. Means ± SDs are presented for each group. *n* = 3. ^*^ *p* < 0.05, ^**^ *p* < 0.01 and ^***^ *p* < 0.001.

TLR4 signaling is involved in the innate immune response elicited by most microbiota. RNA-seq analysis showed that TLR4 signaling pathway–related genes were increased in SPF piglets, especially in JPPs (Figure 6A). We then stimulated Hmy2. cir cells (a human B lymphoblastoid cell line) using common microbial surface antigens (LPS) or major metabolites (SCFAs; acetic acid, propionic acid, and butyric acid) to validate the effect of the commensal microbiota on the TLR4 signaling pathway *in vitro*. The treatment time of 48 h was chosen for these experiments through cell viability and cytotoxicity studies (Supplementary Figure S2). Compared with the control treatment, LPS/SCFA significantly increased the expression of TLR4 signaling pathway-related genes, including *TLR4*, *Myd88*, *IRAK1*, *IRAK4*, *TRAF6*, *TAKA1*, *CHUK*, *IKBKB*, *IL1β*, *IL6*, *TNFα* and *IFNβ1* (Figure 6B, *p* < 0.01), and promoted the secretion of IL6 and TNFα to a certain extent (Figure 6C). These results indicate that the TLR4 signaling pathway is induced in response to microbial colonization.

### 3.6 Commensal microbiota regulates lncRNA expression in piglet PPs

**FIGURE 7:**
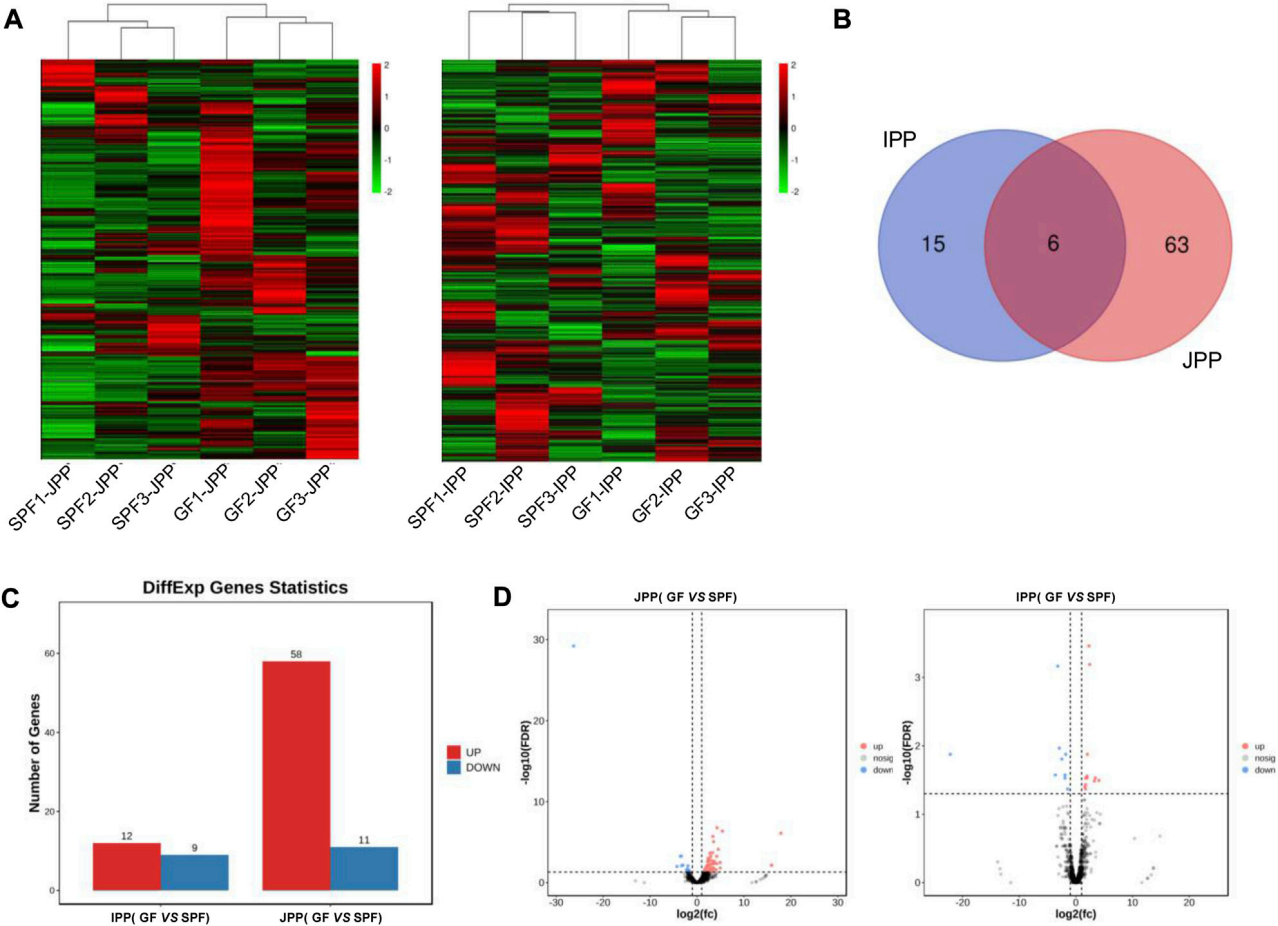
Commensal microbiota regulates lncRNA expression in piglet PPs. **(A)** Hierarchical clustering based on global expression profiles of lncRNAs in IPPs or JPPs. **(B)** The shared and specific DE lncRNAs in IPPs and JPPs. **(C)** The numbers of upregulated and downregulated DE lncRNAs in JPPs and IPPs. **(D)** The DE lncRNAs in JPPs and IPPs were analyzed and visualized *via* volcano plotting. *n* = 3.

LncRNAs exert important regulatory effects on gene expression at the post-transcriptional level. We identified 3,389 substantially expressed lncRNAs (TPM >0.1 in at least three replicates of one group). Hierarchical clustering derived from the global expression of lncRNAs showed a good biological replication in JPPs and IPPs (Figure 7A). We identified 84 DE lncRNAs in twelve samples (SPF vs. GF) with the cutoff |log2(FC)| > 1; FDR <0.01 (Figure 7B). A total of 69 (58 upregulated and 11 downregulated) and 21 (12 upregulated and 9 downregulated) DE lncRNAs were identified in JPPs and IPPs, respectively (Figures 7C, D; Supplementary Table S5). To predict the biological function of DE lncRNAs, *trans*/*cis* analyses were performed to identify highly correlated (|*r*| > 0.9, *p* < 0.05; Supplementary Table S6-1) and locus-adjacent mRNAs (within 100 kb of DE lncRNAs; Supplementary Table S6-2), which showed that 9 (4) and 40 (3) DE mRNAs were highly correlated with and locus-adjacent to DE lncRNAs in JPPs (IPP), respectively (Table 1). We next identified four one-to-one lncRNA–mRNA pairs <100 kb apart that positively correlated with expression levels in JPPs (no lncRNA–mRNA pairs were observed in IPPs) (Supplementary Table S6-3). Intriguingly, some mRNAs, such as *EP300* and *GCNT1,* were closely related to B cell functions. These identified lncRNA–mRNA pairs might mediate the regulatory effects of the commensal microbiota on host intestinal mucosal immunity.

**TABLE 1:** Statistics of *trans* and *cis* analysis [DE lncRNA-mRNA (DE mRNA)].

Tissues	JPP	IPP
*Tans* (|*r*| > 0.90)	47–52 (9)	50-46 (4)
*Cis* (within 100 kb)	69–157 (40)	7-21 (3)
Overlaping of *trans* and *cis*	14–21 (5)	5-7 (0)
lncRNA-mRNA pairs (1:1)	4–4 (4)	0-0 (0)

### 3.7 Gene expression validation by RT-qPCR

**FIGURE 8:**
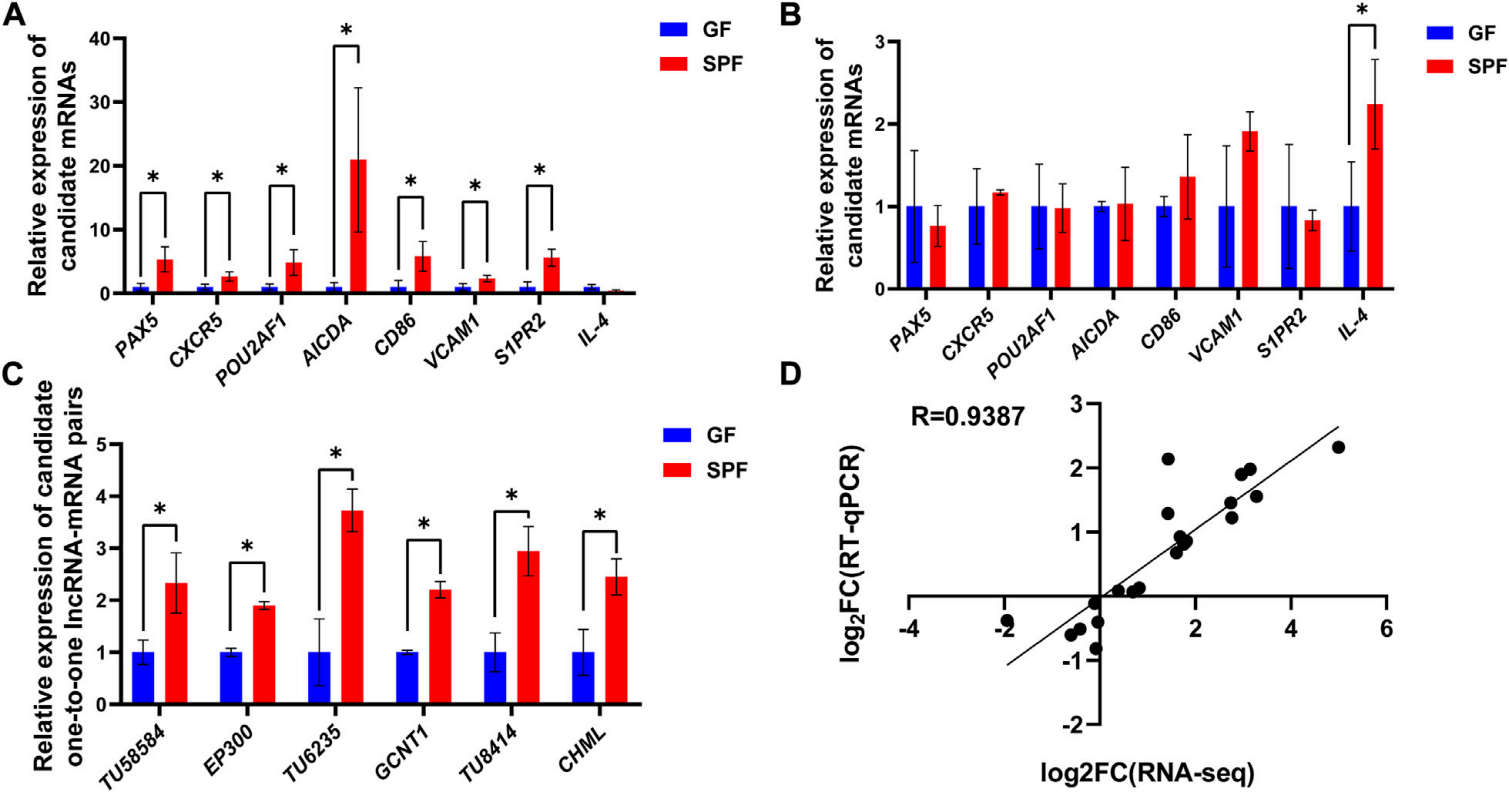
Gene expression validation by RT-qPCR. The relative expression levels of candidate genes in JPPs **(A)** and IPPs **(B)** were detected by RT-qPCR. **(C)** The relative expression levels of lncRNA–mRNA pairs in JPPs. **(D)** Correlation analysis between RNA-seq and RT-qPCR results. Means ± SDs are presented for each group. *n* = 3. ^*^ *p* < 0.05.

The expression levels of candidate genes in SPF and GF piglet PPs were analyzed by RT-qPCR to validate the accuracy of the RNA-seq data. Compared with in GF piglets, in SPF piglets, the expression levels of all B cell function-related genes (*PAX5*, *CXCR5*, *POU2AF1*, *AICDA*, *CD86*, *VCAM1* and *S1PR2*), except *IL-4,* were significantly increased in JPPs (Figure 8A) (*p* < 0.05) but not obviously altered in IPPs (Figure 8B). The expression levels of three one-to-one lncRNA–mRNA pairs (*TU58540*-*EP300*, *TU6235*-*GCNT1* and *TU8414*-*CHML*) were significantly higher in SPF piglet JPPs than in GF piglet JPPs (Figure 8C). The correlation analysis of the above candidate genes showed a high correlation between the RNA-seq and RT-qPCR results (*R* = 0.9387, Figure 8D).

## 4 Discussion

Birth and weaning accelerate animal immune system development due to exposure to the environment and food antigens. Deletion of the microbiota leads to defects in the immune system and incomplete immune cell development (Macpherson and Emma, 2007; Meurens et al., 2007; Elson and Alexander, 2015). PPs play an important role in inducing mucosal immune responses in the gastrointestinal tract. Therefore, this study compared the role of the commensal microbiota in PPs from phenotypic features to gene expression using SPF and GF piglets. First, we found that JPP appearance was more pronounced in SPF piglets, with over 10 JPPs visible to the naked eye on the jejunal wall, while only 5–6 JPPs were visible in GF piglets. IPP appearance on the ileal wall of GF piglets was not obvious. These results were similar to those obtained in previous studies; however, the difference in IPP length between SPF and GF piglets was not significant. The IPP length in previous reports was approximately 100 cm, but approximately 40 cm in this study (data not shown), which might be related to the breed, age, nutritional status, and rearing environment of the piglets (Liebler-Tenorio and Pabst, 2006). Moreover, PPs demonstrated typical histological structures, including the FOR, SED, and IFR, in the absence of the commensal microbiota. The diameter and area of the FOR were significantly larger in SPF piglets than in GF piglets, and those in IPPs were significantly larger than those in JPPs, suggesting that the commensal microbiota, instead of organogenesis, influenced PP development (Sinkora et al., 2011). In addition, a wider IFR was observed in JPPs with a smaller and pear-shaped FOR, whereas a narrower IFR and longer FOR was observed in IPPs, consistent with previous findings (ZhaXi et al., 2014). Furthermore, B/T-cell analysis using pig-specific markers revealed the important role of the commensal microbiota in the immune cell–related development of host intestinal mucosal immunity. These analyses also confirmed that the GC of IPPs is the main site of B-cell development, maturation and response to microbial stimulation (Sinkora et al., 2011).

RNA-seq was used to analyze commensal microbiota-induced transcriptome changes in PPs. Many DE mRNAs between SPF and GF piglet PPs were observed, illustrating that the commensal microbiota affected gene expression in piglet PPs, consistent with previous findings in GF mice (Zdf et al., 2021). Interestingly, the functional enrichment analysis showed that the shared DE mRNAs were mainly involved in basic cellular physiological and metabolic processes. JPP-specific DE mRNAs were involved in immune cell activation and signaling pathways, and IPP-specific DE mRNAs were involved in microbial immune responses, highlighting the common and specific effects of the commensal microbiota on host intestinal mucosal immunity. A previous study revealed the importance of the commensal microbiota for recruiting intestinal lymphocytes by analyzing the expression of chemokines and their receptors in different regions of the small intestine using RT–qPCR (Meurens et al., 2007); the current study also showed that the upregulated mRNAs in both IPPs and JPPs were enriched in cytokine-/chemokine-/receptor-related signaling pathways. Further, we found that the expression levels of B cell function-related genes in GF piglet JPPs were significantly lower than those in SPF piglet PPs. IRF4 is an important factor regulating plasma cell differentiation, and BCL6 inhibits BLIMP1 and favors the expression of genes involved in the B cell maturation in GCs; IL-21 affects plasma cell differentiation (Deng et al., 2015). Both BCL6 and IFR4 were significantly upregulated in SPF piglet JPPs in this study, consistent with previous findings indicating that GC development (especially in B cells) is incomplete in the PPs of germ-free animals (Liu et al., 2020). However, this phenomenon was not obvious in IPPs, similar to the phenotypic analysis findings. Interestingly, the commensal microbiota induced significant differences in the expression levels of some important node genes in specific signaling pathways, such as the intestinal immune network for IgA production (23 and 15 upregulated genes in JPPs and IPPs, respectively, in SPF piglets), suggesting an important role for the commensal microbiota in the induction of host intestinal mucosal immunity and IgA production (Rothkötter, 2009).

LncRNAs, a class of non-coding RNA longer than 200 nt, are emerging as key regulators of various biological processes (Shih and Kung, 2017). This study analyzed the expression patterns of mRNAs and lncRNAs and further explored the relationship between lncRNAs and mRNAs by *trans* (expression level) and *cis* (genomic location) analyses. We identified four lncRNA–mRNA pairs that might mediate the effects of the commensal microbiota on the development of intestinal mucosal immunity. For instance, the EP300–ZNF384 fusion gene product can upregulate GATA3 expression and induce the development and maturation of B cells (Yaguchi et al., 2017). O-glycan remodeling is a feature of B cell differentiation (Giovannone et al., 2018). GCNT1 is reported to cooperate with ST3Gal1 to regulate O-glycan remodeling, ultimately resulting in the expression of distinct O-glycosylation states/CD45 glycoforms during B cell differentiation (Giovannone et al., 2018). In addition, SEC14L1 affects innate immune processes by negatively regulating RIG-I-mediated antiviral signaling (Li et al., 2013) and is involved in the immune process of some diseases, such as lymphovascular invasion in breast cancer (Sonbul et al., 2018). Therefore, we inferred that these identified lncRNA–mRNA pairs may be key mediators of the effect of the commensal microbiota during the development of host PPs and have important guiding significance for the study of host-microbe interaction in the context of mucosal immunity development. Future studies are required for further functional validation of these regulatory axes.

Activating the TLR4 signaling pathway induces the production of many inflammatory factors and chemokines after stimulation with foreign antigen–like microbes (Wang et al., 2020). In this study, the genes involved in the TLR4 signaling pathway were significantly increased in PPs, especially JPPs, in the presence of the commensal microbiota. Similarly, TLR4 signaling pathway-related genes were activated in the human B cell line stimulated with LPS or SCFAs. This result highlighted the crucial role of the TLR4 signaling pathway in the host response to commensal microbiota and their derivatives (Liu et al., 2015).

This study has some limitations that can be addressed in future studies. First, the developmental status of PPs varies in different stages, and comparing the developmental status of PPs at different stages in SPF and GF piglets will help decipher the development of PPs driven by intrinsic (genetic programs) and extrinsic (commensal microbiota) cues. Moreover, only bulk transcriptomic analyses were performed in the present study. Single-cell transcriptome sequencing (scRNA-seq) can be utilized in the future to deeply dissect the cellular mechanisms by which the commensal microbiota influence host intestinal mucosal immunity at high resolution.

## 5 Conclusion

Overall, the findings presented here comprehensively revealed the phenotypic-to-transcriptomic changes in small-intestine PPs in SPF and GF piglets and the effect of the commensal microbiota on piglet PPs development. We also found some important lncRNA–mRNA pairs that potentially mediate the effect of the commensal microbiota during the development of host intestine mucosal immunity.

## Data Availability

The datasets presented in this study can be found in online repositories. The names of the repository/repositories and accession number(s) can be found in the article/Supplementary Material.

## Abbreviations

PPs, Peyer’s patches; GF, germ-free; SPF, specific pathogen-free; JPP, jejunal PP; IPP, ileal PP; DE, differentially expressed; FOR, follicular region; GALT, gut-associated lymphoid tissue; IFR, interfollicular region; IgA, immunoglobulin A; SIgA, secretory IgA; GC, germinal center; GO, Gene ontology; KEGG, Kyoto Encyclopedia of Genes and Genomes; OD, optical density; TPM, Transcripts Per Kilobase of exon model per Million mapped reads; FC, fold change; GSA, Genome Sequence Archive; SD, standard deviation; LPS, lipopolysaccharides; SCFA, short-chain fatty acids; RT-qPCR, Real-time quantitative PCR; CCK8, cell counting kit-8; LDH, lactate dehydrogenase; IOD, integrated optical density.
